# Plasma Exchange in the Management of Catastrophic Antiphospholipid Syndrome

**DOI:** 10.1155/2016/5375080

**Published:** 2016-10-19

**Authors:** Dimitri Titeca-Beauport, Valery Salle, Loay Kontar, Julien Maizel, Gabriel Choukroun

**Affiliations:** ^1^Medical Intensive Care Unit, Amiens University Medical Center, Amiens, France; ^2^Nephrology, Dialysis and Transplantation Department, Amiens University Medical Center, Amiens, France; ^3^Department of Internal Medicine, Amiens University Medical Center, Amiens, France

## Abstract

*Objective*. Report of a case of catastrophic antiphospholipid syndrome (CAPS) with multiple organ involvement leading to a life-threatening condition despite early combination corticosteroid and heparin therapy. Initiation of plasma exchange led to rapid improvement of the patient's general condition.* Design*. Case report.* Setting*. University teaching hospital medical intensive care unit.* Patient*. Single case: 52-year-old man hospitalized for catastrophic antiphospholipid syndrome (CAPS) with cardiac, renal, and cutaneous involvement. Despite early methylprednisolone and heparin therapy, the patient's condition progressively deteriorated, resulting in acute renal failure, right adrenal hemorrhage, and pulmonary involvement, leading to acute respiratory distress on day 6, requiring high-flow nasal cannula oxygen therapy with FiO_2_ of 1.0.* Interventions*. Plasma exchange was started on day 6.* Endpoints and Main Results*. A marked improvement of the patient's general condition was observed after initiation of plasma exchange, with successful weaning of oxygen therapy and normalization of platelet count, troponin, and serum creatinine within four days.* Conclusions*. This case illustrates the efficacy of plasma exchange in CAPS and the difficulty for physicians to determine the optimal timing of plasma exchange.

## 1. Introduction

Antiphospholipid syndrome (APS) is a systemic autoimmune disorder characterized by a combination of arterial and/or venous thrombosis, obstetric complications, and anti-phospholipid antibodies (aPL). The clinical features of APS can range from moderate symptoms to a life-threatening variant called catastrophic antiphospholipid syndrome (CAPS), first described by Asherson in 1992 [1]. CAPS is defined by the involvement of at least three different organ systems over a period of days or weeks and laboratory confirmation of the presence of anti-phospholipid antibodies (aPL) such as anti-*β*_2_-glycoprotein-I (a*β*2GPI) and anti-cardiolipin antibodies (aCL) [2].

## 2. Case Report

We report the case of a 52-year-old man with primary antiphospholipid syndrome (APS) for twelve years, with one episode of deep vein thrombosis, migraine, and persistent high titers of IgG anti-cardiolipin and a*β*2GPI antibodies.

Three weeks before admission to hospital, the patient presented an episode of acute bacterial prostatitis treated by ciprofloxacin for 2 weeks.

On the day of admission, he presented deterioration of his general condition, with weight loss, splinter hemorrhages, leg ulcers, and livedo reticularis.

Electrocardiography (ECG) revealed ST-segment depression in the lateral territory. Echocardiography showed left ventricular dysfunction with an ejection fraction between 30 and 35% but no regional wall motion abnormalities.

Laboratory results showed elevated cardiac troponin at 16.3 *μ*g/L, thrombocytopenia with a platelet count of 76 × 10^3^/mm^3^, no schizocytes, elevated C-reactive protein of 94 mg/L, prothrombin time within the therapeutic range (INR: 4.35), microscopic hematuria without renal failure, and high aPL titers (aCL IgG: 76.7 GPL, IgM: 17.4 MPL, a*β*2GPI IgG: 83.2 SGU, and IgM: 30.8 SMU).

Therapy for catastrophic antiphospholipid syndrome was immediately initiated, comprising three 1 g pulses of methylprednisolone followed by prednisone 80 mg/day and unfractionated heparin (UFH) targeting serum UFH level between 0.3 and 0.7 IU/mL.

Despite treatment, platelet count decreased to 16 × 10^3^/mm^3^ on day 3. Heparin-induced thrombocytopenia was unlikely because of negative ELISA test for heparin-PF4 antibodies.

On day 4, the patient developed acute hypoxemic respiratory failure. Doppler ultrasound did not reveal any signs of deep vein thrombosis. CT scan did not visualize any pulmonary opacities and lung perfusion scintigraphy showed diffuse defects but no arguments in favor of pulmonary embolism. He was admitted to our medical intensive care unit with severe hypoxemia requiring high-flow nasal cannula oxygen therapy with a gas flow of 60 liters per minute and a FiO_2_ of 1.0 to achieve a PaO_2_/FiO_2_ ratio of 81.

**Figure 1:**
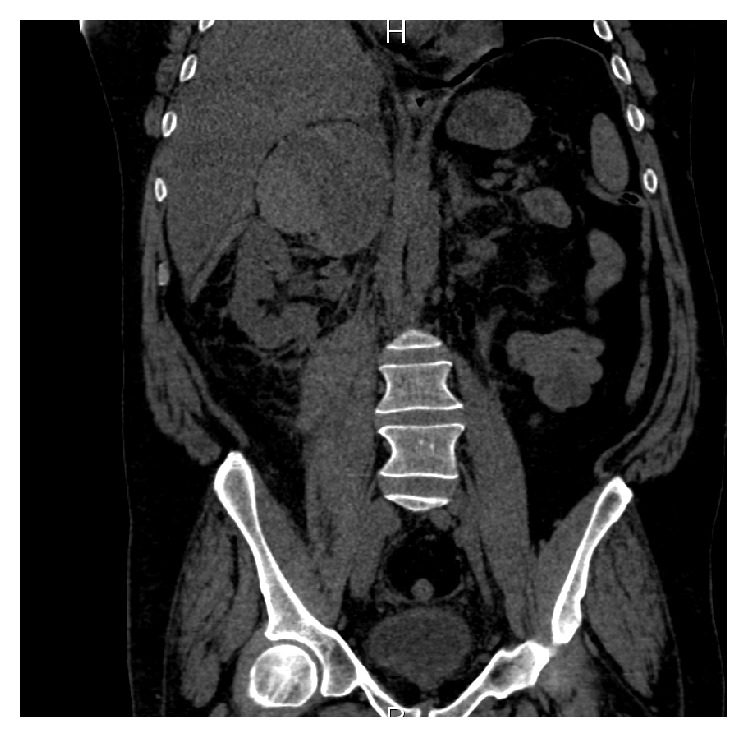
CT scan showing massive right adrenal hemorrhage.

On day 5, he developed right flank pain and another CT scan showed massive right adrenal hemorrhage (Figure 1). He also developed acute renal failure with serum creatinine increased to 246 *μ*mol/L. Laboratory results showed increasing aPL titers with aCL IgG: 147.7 GPL, IgM: 81.4 MPL, a*β*2GPI IgG: 124.8 SGU, and IgM: 148.2 SMU.

**Figure 2:**
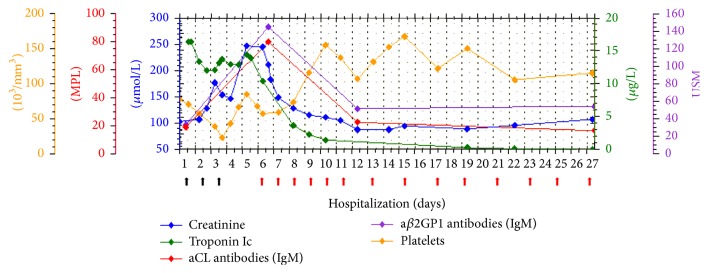
Improvement in laboratory parameters upon initiation of therapeutic plasma exchange. Plasma exchange was initiated on hospital day 6 (red arrows). Methylprednisolone pulses (black arrows).

In view of the patient's worsening general condition associated with an inflammatory syndrome, systemic antibiotic therapy with piperacillin/tazobactam and amikacin was initiated. Unfractionated heparin (UFH) was continued despite the bleeding and daily plasma exchange (PE) was started on day 6. The patient's general condition rapidly improved following initiation of plasma exchange with successful weaning of oxygen and normalization of platelet count, troponin, and serum creatinine within four days (Figure 2). Six days after initiation of PE, laboratory results showed a decline in aPL titers (aCL IgG: 95.8 GPL, IgM: 21.1 MPL, a*β*2GPI IgG: 110.1 SGU, and IgM: 50 SMU).

On day 11, the patient was discharged from the intensive care unit to the medical ward, where plasma exchange was gradually performed less frequently.

## 3. Discussion

This case illustrates the difficulty for the clinicians to evaluate the optimal timing for initiation of PE in patients with CAPS.

Patients with CAPS represent less than 1% of all patients with APS, but CAPS constitutes the first manifestation of APS in approximately one-half of cases. More than one-third of cases of CAPS are triggered by acute infection [3]. This syndrome is also characterized by higher plasmatic ferritin concentration compared to patients with classic APS, which could contribute to the “cytokine storm,” leading to multiple organ dysfunction [4]. In this regard, some authors proposed to include CAPS in a common syndrome entity termed “Hyperferritinemia Syndrome” including macrophage activation syndrome, adult onset Still's disease, and septic shock [5].

CAPS is a life-threatening condition that usually requires admission to the ICU [6]. In a published series of 250 cases from 1992 to 2005, the mortality rate was 48%. Cerebral complications, cardiac complications, and infections were the main causes of death. The only independent poor prognostic factor was the association with systemic lupus erythematosus (SLE). In this series, the most commonly reported treatment combination was anticoagulants (AC) plus corticosteroids (CS) with a recovery rate of only 63.8%. The highest recovery rate (77.8%) was achieved with a combination of AC + CS + PE, followed by AC + CS + PE and/or IVIG (69%) [5]. Since 2001, the increased use of AC + CS + PE and/or IVIG has resulted in a 20% reduction of mortality, with a current mortality rate of 39% [3, 7]. In a series of 230 cases of CAPS, treatment with PE was independently associated with decreased mortality [OR 0.36 (0.14–0.92) *p* = 0.033] [6]. PE therefore appears to be the most effective treatment for CAPS, probably through prompt removal of circulating anti-phospholipid antibodies [8–10]. We may also consider that PE could remove proinflammatory mediators such as serum ferritin, TNF-*α*, IL-1, IL-6, and IL-18 [5].

The place of immunosuppressant therapy in CAPS has not been clearly defined. Cyclophosphamide was used in one-third of cases but has only been shown to be beneficial in the case of SLE flare [8]. Rituximab appeared to be beneficial in a series of twenty cases with a recovery rate of 75% and Eculizumab use has been reported in some refractory cases of CAPS [11].

In this case of CAPS with multiorgan involvement, PE was delayed due to the initial absence of any clinical signs of severity. Despite corticosteroid and unfractionated heparin therapy, the patient's general condition progressively deteriorated until initiation of PE.

Guidelines from international consensus statements recommend initiation of PE in the case of life-threating forms of CAPS (especially in the presence of schizocytes) [12]. On the other hand, only 30% of more than 400 cases reported by the CAPS registry received PE [3]. PE therefore does not appear to be always necessary in patients with CAPS. More prognostic factors must be defined to help clinicians identify those patients who will require early initiation of PE.

The major challenge therefore consists of identifying those patients likely to benefit from this therapy and determining the optimal timing of initiation of PE. Monitoring of laboratory parameters, such as increased aPL titers, troponin, serum creatinine, and decreased platelet count can contribute to evaluation of the efficacy of treatment and decision-making. In the case reported here, PE was rapidly effective, probably because of the high aPL titers [13].

## 4. Conclusion

CAPS is an uncommon but potentially life-threatening condition. In view of the lack of clinical trials, optimal management remains unknown, but a combination of AC + CS + PE and/or IVIG appears to be the most effective treatment option. This case illustrates the difficulty for physicians to determine the optimal timing of plasma exchange. A better understanding of the pathophysiology and a more precise description of clinical and laboratory characteristics could help clinicians identify those patients who may benefit from early initiation of PE.
